# Promoting cemented fixation of the femoral stem in elderly female hip arthroplasty patients and elderly hip fracture patients: a retrospective cohort study from the Norwegian Arthroplasty Register and the Norwegian Hip Fracture Register 

**DOI:** 10.2340/17453674.2024.40073

**Published:** 2024-02-23

**Authors:** Jan-Erik GJERTSEN, Daniel NILSEN, Ove FURNES, Geir HALLAN, Gard KROKEN, Eva DYBVIK, Anne Marie FENSTAD

**Affiliations:** 1 The Norwegian Hip Fracture Register, Department of Orthopaedic Surgery, Haukeland University Hospital, Bergen; 2 Department of Clinical Medicine, University of Bergen, Bergen; 3 The Norwegian Arthroplasty Register, Department of Orthopaedic Surgery, Haukeland University Hospital, Bergen; 4 Directorate of Fisheries, Section for Analysis and Risk Assessment, Bergen, Norway

## Abstract

**Background and purpose:**

Uncemented stems increase the risk of revision in elderly patients. In 2018, we initiated a national quality improvement project aiming to increase the proportion of cemented stems in elderly female total hip arthroplasty (THA) and hip fracture hemiarthroplasty (HA) patients. We aimed to evaluate the association of this project on the frequency of cemented stems and the risk of secondary procedures in the targeted population.

**Methods:**

10,815 THAs in female patients ≥ 75 years in the Norwegian Arthroplasty Register and 19,017 HAs in hip fracture patients ≥ 70 years in the Norwegian Hip Fracture Register performed in 2015–2017 and 2019–2021 at all Norwegian hospitals were included in this retrospective cohort study. The quality improvement project was implemented at 19 hospitals (8,443 patients). 1-year revision risk (THAs) and reoperation risk (HAs) were calculated for uncemented and cemented stems by Kaplan–Meier and Cox adjusted hazard rate ratios (aHRRs) with all-cause revision/reoperation as main endpoint.

**Results:**

The use of cemented stem fixation in the targeted population increased from 26% to 80% for THAs and from 27% to 91% for HAs. For THAs, the 1-year revision rate decreased from 3.7% in 2015–2017 to 2.1% in 2019–2021 (aHRR 0.7, 95% confidence interval [CI] 0.5–0.9) at the intervention hospitals. For HAs, the reoperation rate decreased from 5.9% in 2015–2017 to 3.3% in 2019–2021 (aHRR 0.6, CI 0.4–0.8) at the intervention hospitals.

**Conclusion:**

The quality improvement project resulted in a significant increase in the proportion of cemented stems and reduced risk of secondary procedures for both THAs and HAs.

Each year approximately 10,000 patients are treated with a primary total hip arthroplasty (THA) and 3,500 hip fracture patients are treated with a hemiarthroplasty (HA) in Norway [1]. A large Nordic multinational register study showed that cemented implants had better survival than uncemented implants in patients aged 65 years or older, while no such differences could be found in younger patients [2]. An earlier study from the Norwegian Arthroplasty Register (NAR) reported higher risk of revision for uncemented stems in women aged 55 years or older and advised against use of uncemented stems in these patients [3]. Studies have suggested that the risk of periprosthetic femoral fracture (PPFF) is high after THAs with an uncemented stem, particularly in the oldest patients and in women [2-8]. Similarly, the risk for revision among HA patients, in particular due to PPFF, has been found to be higher when using an uncemented femoral stem [9-12]. A revision of a THA or a HA due to a PPFF represents a serious adverse event associated with high mortality and reduced functional outcome [13-15]. International guidelines advocate use of cemented stems when treating hip fracture patients [16-18].

Nevertheless, a paradoxical increase in the use of uncemented stems was identified in Norway before this quality improvement project was initiated [1]. There are several probable causes. From a health-economic perspective, one can argue that an uncemented prosthesis has shorter operating time than a cemented prosthesis. Reluctance to use cemented stems may also be due to fear of bone cement implantation syndrome [19]. For hip fracture patients, treatment with a cemented stem has been found to increase mortality in the first 2 postoperative days compared with treatment with an uncemented stem, but not after 1 year [11,20-22]. Nor has a clinically relevant difference in mortality been reported between uncemented and cemented THAs for osteoarthritis [23]. In 2018, based on a concern over increased use of uncemented stems, the NAR and the Norwegian Hip Fracture Register (NHFR) initiated a national quality improvement project promoting cemented fixation of the femoral stem in elderly female hip arthroplasty patients and hip fracture patients. We aimed to investigate the association of this national quality improvement project on the frequency of cemented stems used and on the risk of secondary procedures in the targeted population.

## Methods

### Study setup

This is a retrospective observational study based on prospectively collected data from the NAR and the NHFR. The goal was, at a national level, to use cemented stems in more than 90% of female THA patients ≥ 75 years and in more than 95% of hip fracture HA patients ≥ 70 years. We reported according to the STROBE statement.

### The Norwegian Arthroplasty Register

The NAR has registered detailed information on primary THAs and revision THAs performed in Norway since 1987 [**24**]. After each primary operation and revision, the surgeon register data which is sent to the NAR. The dataset includes patient information (age, sex, and comorbidity [ASA classification]), the date and indication for surgery, and detailed information on the type and fixation of the implant. Revisions, defined as any secondary procedure involving exchange or removal of one or more prosthesis components, are linked to the primary operation with use of the 11-digit Norwegian personal identification number. The completeness of reporting to the NAR is 97% for primary THAs and 91% for THA revisions [1].

### The Norwegian Hip Fracture Register

The NHFR has registered detailed information on hip fracture surgery in Norway since 2005 [25]. As for the NAR, the surgeon registers data after each primary operation and reoperation. The dataset includes patient information (age, sex, comorbidity [ASA classification]), and cognitive status), information on time and type of fracture, the time and type of surgery, and detailed information on the type and fixation of the implant. Reoperations, defined as any secondary procedure, including osteosynthesis for PPFF and soft-tissue debridement for infection, are linked to the primary operation with use of the 11-digit Norwegian personal identification number. The completeness of reporting to the NHFR is 92% for primary HAs and 88% for HA reoperations [1].

### Implementation of the quality improvement project

The intervention included 15 hospitals using 50% or fewer cemented femoral stems in female THA patients > 75 years who were invited to participate in the THA project. Of these hospitals, 9 also used a low proportion (< 40%) of cemented HAs and were invited to participate in the HA project. In addition, 4 hospitals with a higher proportion of cemented femoral stems decided to participate in the project. At the start-up seminar in October 2018 the scientific evidence supporting the recommendation to use cemented stems in the relevant patient categories was presented. The status at each intervention hospital was reviewed and discussed at 4 follow-up seminars. Several obstacles at the hospitals were identified, including fear of bone cement implantation syndrome, scant experience with cementing of femoral stems, and uncertainty concerning which prosthesis stem to choose. In addition, the intervention hospitals were regularly contacted by email or by phone between the seminars.

### Statistics

Continuous data was described using means and standard deviation (SD). All analyses were performed separately for THAs and HAs. Annual proportions of cemented stems used in THAs and HAs were calculated for the included hospitals and for all hospitals reporting to the NAR and NHFR respectively. Implant survival of all stems, all uncemented stems, and all cemented stems in 2 different time-periods (2015–2017 and 2019–20^21^) at all participating hospitals and at all hospitals was calculated using Kaplan–Meier analyses with revision as endpoint for THAs and reoperation as endpoint for HAs. Adjusted hazard rate ratios (aHRRs) for 1-year revision/reoperation for any cause and for 1-year revision/reoperation due to PPFF were calculated using Cox regression analyses, comparing the 2 different time periods with 2015–2017 as reference. Further, aHRRs for revision/reoperation for any cause and for revision/reoperation due to PPFF were calculated for uncemented and cemented stems in the 2 time periods with uncemented stems 2015–2017 as reference. We used the free program DAGitty (www.dagitty.net) version 3.1 (2023) to verify variables that needed to be adjusted for in the 2 Cox models. We developed directed acyclic graphs (DAGs) for revision of THAs and reoperation of HAs. The HRRs for the exposures (cemented/uncemented stems) were adjusted according to this model (THAs: age, ASA class, diagnosis, and surgical approach. HAs: age, sex, ASA class, cognitive function, fracture type, and surgical approach). Bilateral THAs and HAs are dependent observations. However, the proportions of bilateral operations in this study were low (7.1% and 3.7% respectively). The influence of bilaterality on the outcome has earlier been found to be negligible and hence the bilateral operations were evaluated independently [26]. Mean (SD) follow-up time for THAs was 5.9 (1.5) years in 2015–2017 and 2.4 (0.9) years in 2019–2021. For HAs, mean follow-up time was 3.4 (2.5) and 1.7 (1.9) for 2015–2017 and 2019–2021 respectively. Patients were followed from primary operation to revision/reoperation, death or until December 31, 2022, whichever occurred first. Mortality data was collected from the Norwegian Population Registry. Accordingly, all prostheses had a minimum of 1 year follow-up. The proportional hazard assumption for the Cox model was tested based on Schoenfeld residuals and found to be fulfilled. HRRs are presented with 95% confidence intervals (CIs). The statistical analyses were performed using IBM SPSS Statistics 29.0 (IBM Corp, Armonk, NY, USA) and the STATA 17 (StataCorp LLC, College Station, TX, USA, 2021).

### Ethics, data sharing plan, funding, and disclosures

The NAR and NHFR have licenses from the Norwegian Data Protection Authority (reference numbers 03/00058-15/JTA [last issued on January 24, 2017] and 2004/1658-2 SVE/- [issued on January 3, 2005]) respectively. The regulations of the Norwegian Data Protection Authority and the Norwegian personal protection laws prohibit the publication of the complete dataset. The NAR and the NHFR is financed by the Western Norway Regional Health Authority. The quality improvement project was funded by the Norwegian Advisory Unit for Medical Quality Registries, Northern Norway Regional Health Authority, Tromsø, Norway. The Regional Centre for Clinical Quality Registries, Western Norway Regional Health Authority, Bergen, Norway awarded a grant to the first author to write this article. JEG and GH have received speaker fees from Ortomedic (Norwegian distributor for DePuy Synthes) and LINK Norway. The employer of OF has received fees for lectures on cementing technique for knee replacement given by Heraeus Medical and Ortomedic. Complete disclosure of interest forms according to ICMJE are available on the article page, doi: 10.2340/17453674.2024.40073

## Results

**Figure 1:**
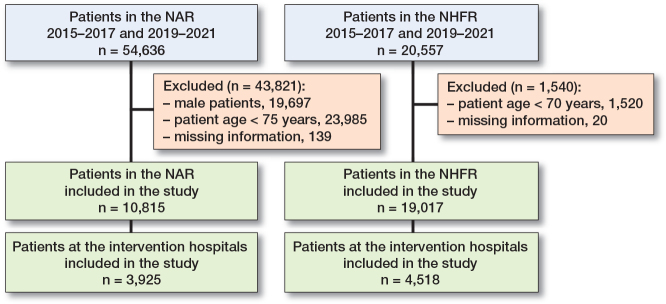
Flowchart of patient

Patients operated on during the 3 years before (2015–2017) and after (2019–2021) the initiation of the project were included, resulting in 54,636 primary THAs reported to the NAR. We excluded male patients (n = 19,697), patients < 75 years of age (n = 23,985), and patients with missing information on fixation type (n =139). Finally, 10,815 THAs in females ≥ 75 years were included in the analyses. Of these, 3,925 patients were operated on at the intervention hospitals (Figure 1). In the same time periods 20,557 primary HAs were reported to the NHFR. We excluded patients < 70 years of age (n = 1,520) and patients with missing information on fixation type (n = 20). Finally, 19,017 HAs in patients ≥ 70 years were included. Of these, 4,518 HAs were operated on at the intervention hospitals (Figure 1).

### Total hip arthroplasties in women ≥ 75 years

The mean age of the included THA patients was 81 years and 32% of the patients had severe comorbidity (ASA 3–5). Most patients had osteoarthritis as the indication for surgery (84%). A posterior approach was the most frequently used surgical approach (68%) (Table 1).

**Table 1:** Baseline characteristics of primary total hip arthroplasties in periods 2015–2017 and 2019–2021 reported to NAR. Values are frequency (%) unless otherwise specified

Factor	Intervention hospitals	All hospitals
Total, n	3,925	10,815
Mean age (SD)	80.6 (4.2)	80.7 (4.3)
Women	3,925 (100)	10,815 (100)
ASA score		
1–2	2,592 (67)	7,274 (68)
3–5	1,292 (33)	3,450 (32)
missing	41	91
Diagnosis		
osteoarthritis	3,358 (86)	9,084 (84)
rheumatoid arthritis	42 (1.1)	111 (1.0)
sequelae after hip fracture	231 (5.9)	594 (5.5)
acute intracapsular fracture	83 (2.1)	473 (4.4)
other	202 (5.1)	530 (4.9)
missing	9	23
Surgical approach		
anterior	557 (15)	732 (6.8)
anterolateral	855 (23)	1,360 (13)
lateral	349 (8.9)	1,221 (12)
posterior	2,024 (53)	7,094 (68)
missing	140	408

ASA, American Society of Anesthesiologists.

**Figure 2:**
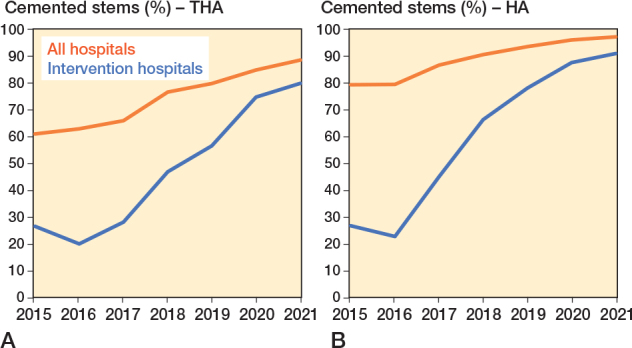
A. Proportion of total hip arthroplasties (THA) with cemented femoral stems in women ≥ 75 years of age reported to NAR. B. Proportion of hemiarthroplasties (HA) with cemented femoral stems in patients ≥ 70 years of age reported to NHFR.

**Figure 3:**
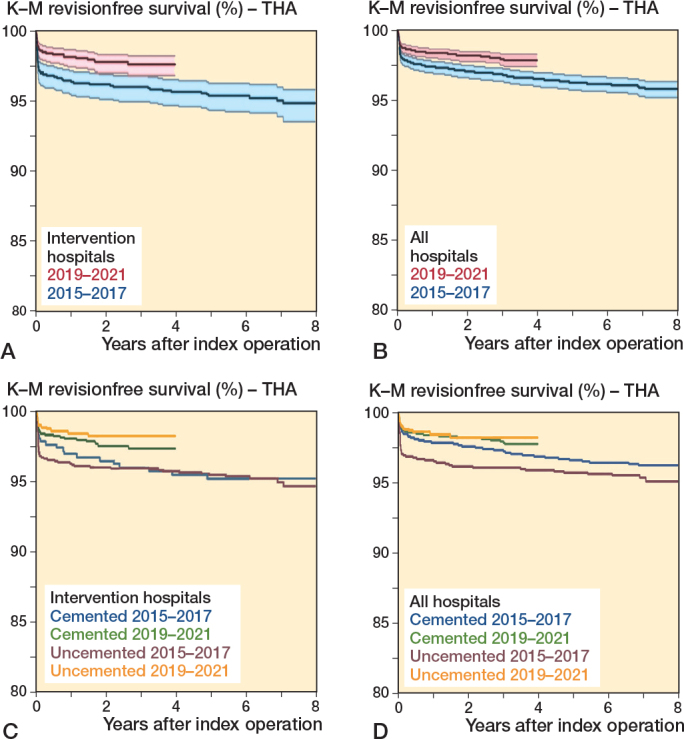
Prosthesis survival of total hip arthroplasties in women ≥ 75 years of age reported to the NAR 2015–2017, and 2019–2021 with revision of any cause as endpoint. A. Intervention hospitals—all revisions (log rank P = 0.01). B. All hospitals—all revisions (log rank P < 0.001). C. Intervention hospitals—cemented vs. uncemented stems in the 2 time periods—all revisions (log rank P = 0.03). D. All hospitals—cemented vs. uncemented stems in the 2 time periods—all revisions (log rank P < 0.001).

For intervention hospitals, the proportion of cemented femoral stems increased from 26% in 2015 to 80% in 2021 (Figure 2A). The 1-year implant survival of primary THAs increased from 97.3% (CI 95.5–97.1) in 2015–2017 to 97.9% (CI 97.3–98.5) in 2019–2021 (log rank: P = 0.01) (Figure 3A). In 2019–2021 there was a reduced 1-year risk of all-cause revision compared with 2015–2017 (aHRR 0.7, CI 0.5–0.9) (Table 2). There was also a reduction in revisions due to PPFF from 0.7% to 0.4%, but the reduction was not statistically significant (Table 2). Less pronounced reductions could be found when analyzing the results for all hospitals (Figure 3B and Table 2). Survival curves for uncemented and cemented THAs in the 2 time periods are shown in Figure 3C and Figure 3D. For intervention hospitals, THAs in 2019–2021 had a reduced 1-year risk of all-cause revision compared with THAs with uncemented stem in 2015–2017. Further, cemented THAs in 2019–2021 had a lower risk of PPFF compared with uncemented THAs in 2015–2017 (Table 3). Risk of revisions after THAs with uncemented and cemented stems in the 2 time periods for all hospitals is given in Table 3.

**Table 2:** Number of reoperations and adjusted hazard rate ratios (aHRR) for 1-year revision after total hip arthroplasty

Revisions	2015–2017		2019–2021		aHRR^a^ (CI)
	Total	Revisions	Total	Revisions	
	n	n (%)	n	n (%)	
Intervention hospitals					
All	1,866	69 (3.7)	2,059	43 (2.1)	0.51 (0.32–0.81)
Due to PPFF	1,866	13 (0.7)	2,059	8 (0.4)	0.66 (0.22–2.0)
All hospitals					
All	5,232	140 (2.7)	5,583	95 (1.7)	0.65 (0.48–0.86)
Due to PPFF	5,232	26 (0.5)	5,583	13 (0.2)	0.64 (0.29–1.4)

PPFF, periprosthetic fracture of the femur.

a Cox regression analysis with adjustments for age, ASA classification, diagnosis, and surgical approach. 2015–2017 is reference.

**Table 3:** Number of revisions and adjusted hazard rate ratios (aHRR) for revision after total hip arthroplasty at participating hospitals and all hospitals in Norway by time period and type of stem fixation

Factor	Total n	Revisions n (%)	aHRR^a^ (CI)
Intervention hospitals—all revisions			
Uncemented 2015–2017	1,407	54 (3.8)	1 Reference
Cemented 2015–2017	459	15 (3.3)	0.63 (0.34–1.2)
Uncemented 2019–2021	613	11 (1.8)	0.34 (0.16–0.72)
Cemented 2019–2021	1,446	32 (2.2)	0.46 (0.28–0.75)
Intervention hospitals—revision due to PPFF			
Uncemented 2015–2017	1,407	12 (0.9)	1 Reference
Cemented 2015–2017	459	1 (0.2)	0.20 (0.03–1.6)
Uncemented 2019–2021	613	5 (0.8)	0.57 (0.15–2.2)
Cemented 2019–2021	1,446	3 (0.2)	0.20 (0.05–0.72)
All hospitals—all revisions			
Uncemented 2015–2017	1,926	68 (3.5)	1 Reference
Cemented 2015–2017	3,306	72 (2.2)	0.55 (0.38–0.78)
Uncemented 2019–2021	874	14 (1.6)	0.33 (0.17–0.65)
Cemented 2019–2021	4,709	81 (1.7)	0.43 (0.30–0.62)
All hospitals—revision due to PPFF			
Uncemented 2015–2017	1,926	19 (1.0)	1 Reference
Cemented 2015–2017	3,306	7 (0.2)	0.16 (0.06–0.46)
Uncemented 2019–2021	874	5 (0.6)	0.39 (0.11–1.4)
Cemented 2019–2021	4,709	8 (0.2)	0.16 (0.07–0.40)

PPFF, periprosthetic fracture of the femur.

a Cox regression analyses: adjustments for age, ASA classification, diagnosis, and surgical approach.

### Hemiarthroplasties in patients ≥ 70 years

The mean age of the patients treated with an HA for acute hip fracture was 84 years, 67% were females, and 71% of the patients had severe comorbidity (ASA 3–5). Most patients had intracapsular femoral neck fracture as indication for surgery (97%). The direct lateral approach was the most frequently used surgical approach (74%) (Table 4).

**Table 4:** Baseline characteristics of primary hemiarthroplasties in periods 2015–2017 and 2019–2021 reported to NHFR. Values are frequency (%) unless otherwise specified

Factor	Intervention hospitals	All hospitals
		
Total, n	4,518	19,017
Mean age (SD)	83.7 (7.0)	83.9 (6.9)
Women	3,028 (67)	12,759 (67)
ASA score		
1–2	1,316 (29)	5,387 (28)
3–5	3,162 (71)	13,452 (71)
missing	40	178
Cognitive function		
chronic cognitive impairment	1,277 (28)	5,595 (29)
uncertain	375 (8.3)	1,545 (8.1)
cognitively fit	2,779 (62)	11,387 (60)
Diagnosis		
acute intracapsular fracture	4,397 (97)	18,533 (97)
acute extracapsular fracture	93 (2.1)	369 (1.9)
other	28 (0.6)	115 (0.6)
Surgical approach		
anterior	47 (1.0)	329 (1.7)
anterolateral	182 (4.0)	1,371 (7.2)
lateral	3,504 (78)	13,939 (74)
posterior	735 (16)	3,106 (17)
missing	50	272

ASA, American Society of Anesthesiologists.

**Figure 4:**
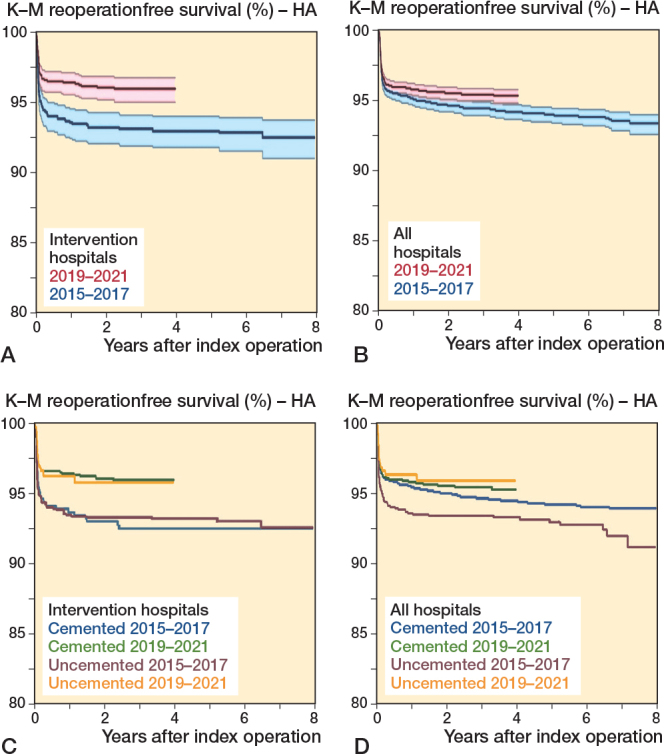
Prosthesis survival of hemiarthroplasties reported to the NHFR 2015–2017 and 2019–2021 with reoperation of any cause as endpoint. A. Intervention hospitals—all reoperations (log rank P < 0.001). B. All hospitals –all reoperations (log rank P = 0.00). C. Intervention hospitals—cemented vs. uncemented stems in the 2 time periods—all reoperations (log rank P < 0.001). D. All hospitals—cemented vs. uncemented stems in the 2 time periods—all reoperations (log rank P < 0.001).

For intervention hospitals, the proportion of cemented HAs increased from 27% in 2015 to 91% in 2021 (Figure 2B). The 1-year implant survival of primary HAs increased from 94.6% (CI 92.4–94.8) in 2015–2017 to 96.4% (CI 95.6–97.2) in 2019–2021 (log rank: P < 0.001) (Figure 4A). In 2019–2021 there was a reduced 1-year risk of all-cause reoperation compared with 2015–2017 (Table 4). There was also a reduction in reoperation due to PPFF from 0.7% to 0.2%, but the reduction was not statistically significant (Table 5). Less pronounced reductions could be found when analyzing the results for all hospitals (Figure 4B and Table 5). Survival curves for uncemented and cemented HAs in the 2 time periods are shown in Figure 4C and Figure 4D. For intervention hospitals no statistically significant difference in 1-year risk of all-cause reoperation could be found between uncemented and cemented HAs 2015–2017 (aHRR 0.9, CI 0.6–1.3). Compared with uncemented HAs 2015–2017, both cemented HAs 2019–2021 (aHRR 0.5, CI 0.4–0.7) and uncemented HAs 2019–2021 (aHRR 0.5, CI 0.3–1.0) had a reduced 1-year risk of all-cause reoperation (Table 6). Including all hospitals, cemented HAs in both time periods and uncemented HAs 2019–2021 had a reduced 1-year risk of all-cause revision compared with uncemented HAs 2015–2017. Further, cemented HAs in both time periods had a lower risk of reoperation due to PPFF than uncemented HAs 2015–2017 (Table 6).

**Table 5:** Number of reoperations and adjusted hazard rate ratios (aHRR) for 1-year reoperation after hemiarthroplasty reported to NHFR

Reoperations	2015–2017		2019–2021		aHRR^a^ (CI)
	Total n	Reop.n (%)	Total n	Reop.n (%)	
Intervention hospitals					
All	2,156	128 (5.9)	2,362	78 (3.3)	0.56 (0.40–0.78)
Due to PPFF	2,156	16 (0.7)	2,362	5 (0.2)	0.50 (0.14–1.8)
All hospitals					
All	9,246	413 (4.5)	9,771	375 (3.8)	0.87 (0.75–1.0)
Due to PPFF	9,246	38 (0.4)	9,771	25(0.3)	0.80 (0.46–1.4)

PPFF, periprosthetic fracture of the femur.

a Cox regression analysis with adjustments for age, sex, ASA classification, fracture type, and surgical approach. 2015–2017 is reference.

**Table 6:** Number of reoperations and adjusted hazard rate ratios (aHRR) for reoperation after hemiarthroplasty at participating hospitals and all hospitals in Norway by time period and type of stem fixation

Factor	Total n	Reop.n (%)	aHRR^a^ (CI)
Intervention hospitals—all reoperations			
Uncemented 2015–2017	1,484	89 (6.0)	1 Reference
Cemented 2015–2017	672	39 (5.8)	0.91 (0.61–1.3)
Uncemented 2019–2021	342	11 (3.2)	0.52 (0.28–0.97)
Cemented 2019–2021	2,020	67 (3.3)	0.52 (0.38–0.72)
Intervention hospitals—reoperation due to PPFF			
Uncemented 2015–2017	1,484	15 (1.0)	1 Reference
Cemented 2015–2017	672	1 (0.2)	0.15 (0.02–1.1)
Uncemented 2019–2021	342	2 (0.6)	0.48 (0.11–2.2)
Cemented 2019–2021	2,020	3 (0.2)	0.08 (0.02–0.36)
All hospitals—all reoperations			
Uncemented 2015–2017	1,694	100 (5.9)	1 Reference
Cemented 2015–2017	7,552	313 (4.1)	0.69 (0.55–0.86)
Uncemented 2019–2021	450	14 (3.1)	0.52 (0.30–0.91)
Cemented 2019–2021	9,321	361 (3.9)	0.63 (0.50–0.78)
All hospitals—reoperation due to PPFF			
Uncemented 2015–2017	1,694	20 (1.2)	1 Reference
Cemented 2015–2017	7,552	18 (0.2)	0.19 (0.10–0.37)
Uncemented 2019–2021	450	2 (0.4)	0.35 (0.08–1.5)
Cemented 2019–2021	9,321	23 (0.2)	0.19 (0.10–0.35)

PPFF, periprosthetic fracture of the femur.

a Cox regression analyses: adjustments for age, sex, ASA classification, fracture type, and surgical approach.

## Discussion

The aim of the study was to investigate the association of a registry-driven national quality improvement project on the frequency of cemented stems used and on the risk of secondary procedures in the targeted population. We showed that during a 7-year period the proportion of cemented stems used in female patients ≥75 years treated with a THA and in hip fracture patients ≥ 70 years treated with an HA increased substantially. The increased use of cemented stems was identified both at intervention hospitals and at a national level. The risk of revision after a primary THA and the risk of reoperation after a primary HA declined substantially in 2019–2021 compared with 2015–2017. At the intervention hospitals, both prostheses with cemented stems and prostheses with uncemented stems in the last period had a lower risk of secondary procedures compared with uncemented prostheses in the first period.

Our findings add additional support to a shift towards cemented stem fixation in the elderly, especially in females.

This register-run project was an effective way of getting hospitals to change their practice. The change in practice also resulted in a significantly increased proportion of cemented stems at a national level and a reduction in the number of secondary procedures. Surprisingly, the difference in reoperation risk between uncemented and cemented stems operated on in the pre-intervention period was small at the participating hospitals. The main reason for this was poorer implant survival for prostheses with cemented stems at that time, assumably due to a strong tradition of using uncemented stems, and good technique when inserting these, together with non-familiarity with cemented stems. Therefore, they had not changed practice before the start of the quality improvement project. A learning curve at the hospitals already in the process of changing to cemented stems during the first period could also have attributed to poorer results with use of cemented stems. In addition, some hospitals changed from an uncemented stem to a cemented polished taper-slip stem, a design also known to have an increased risk of revision due to PPFF [27-28]. In the last period, implant survival was also better for all prostheses at a national level. The number of revisions/reoperations due to PPFF was low for both THAs and HAs. Accordingly, the CIs were wider than for all secondary procedures and no statistically significant reduction could be found. Therefore, generalization of the results of this study should be done with caution.

Even if the change of practice from uncemented to cemented stems had already started before the national quality improvement project was initiated, probably due to publications from our registries [5,10], it seems clear that the project led to a further substantial decrease in the number of uncemented femoral stems used, both in elderly female THA patients and in elderly hip fracture HA patients. The observed change of practice has reduced the risk of secondary procedures at a national level. Any secondary procedure after arthroplasty surgery represents a temporary increase in morbidity and mortality for a patient as well as a risk of a poorer functional result [29-31]. Consequently, this project has made arthroplasty surgery in elderly patients safer in Norway.

### Strengths

The major strength of this quality improvement study was the high number of patients. We were able to include all hospitals using a low proportion of femoral stems in the pre-defined patient categories. Both low-volume and high-volume hospitals were included, and the hospitals represented all 4 regional health authorities in Norway. The completeness of registration in the NAR and NHFR is high for both primary operations and revisions [1]. Unfortunately, we do not know the completeness of reporting for specific revision causes. Presumably completeness and accuracy are poorer for certain reoperation causes, such as infection or periprosthetic fracture with retention of the prostheses. As the quality improvement project was evaluated with registry data, we were able to investigate both changes in clinical practice and changes in outcome after THAs and HAs during the study period.

### Limitations

Residual confounding cannot be eliminated completely. Accordingly, we can show associations, but not prove causality. Even if we observed a significant increase in the use of cemented stems parallel to a reduced risk of reoperations after both THAs and HAs, there may be other reasons for this reduction in reoperations. As hospitals started to use cemented stems more frequently at different times, a learning curve could have been present during the study period. To minimize the influence of changing practice, we chose to exclude operations in 2018 and compared the last 3 years before and the first 3 years after the improvement project was initiated. Finally, we had a short follow-up time. However, previous literature has shown that, with contemporary implants, a large proportion of the secondary procedures occur in the first year postoperatively [22,32].

### Conclusion

This quality improvement project resulted in a significant and desirable increase in the use of cemented stems in both THAs in women ≥ 75 years and in HAs in hip fracture patients ≥ 70 years. This change of practice coincided with a reduced risk of reoperations, which was reduced even at a national level. Using a national registry was a feasible and effective way of conducting a national quality improvement project.

In perspective, this study underlines that quality improvement studies have good potential to change practice and should probably be used more.

## Notes

JEG, DN, OF, GH, ED, and AMF planned the study. JEG wrote the initial draft of the manuscript. AMF and GK performed the statistical analyses. All authors contributed to the interpretation of the findings, and improvement of the manuscript.

The authors thank the Norwegian surgeons participating in the quality improvement project and the Regional Centre for Clinical Quality Registries, Western Norway Regional Health Authority, Bergen, Norway for advice and support in this project.

Handling co-editors: Cecilia Rogmark and Philippe Wagner

*Acta* thanks Martin Lindberg-Larsen and Olof Sköldenberg for help with peer review of this manuscript.
